# Cognitive impairment and depression in a population of patients with chronic kidney disease in Colombia: a prevalence study

**DOI:** 10.1186/s40697-016-0116-7

**Published:** 2016-05-10

**Authors:** Carlos Edgardo Rodríguez-Angarita, Rafael Mauricio Sanabria-Arenas, Juan Diego Vargas–Jaramillo, Izcay Ronderos–Botero

**Affiliations:** Research Group in Neurology and Psychiatry (INEUROPSI), Fundación Universitaria de Ciencias de la Salud (FUCS), Carrera 19 No 8A-32, Bogotá, D.C., Colombia; Renal Teraphy Services (RTS), Bogotá, D.C., Colombia

**Keywords:** Cognitive impairment, Depression, Chronic kidney disease, Aging, Prevalence

## Abstract

**Background:**

Growth of the elderly population is linked to the increase of comorbid conditions such as chronic kidney disease (CKD), depression, and cognitive impairment (CI). Cognitive impairment can vary from minimal deficits in the normal aging, to mild cognitive impairment with a prevalence ranging from 1 to 29 % in people ≥ 65 years of age, up to severe impairment with a prevalence of 6 to 42 %. The CI induced by depression usually affects the functional performance of the elderly.

**Objective:**

The objective of the study is to describe the prevalence of CI and depression in patients ≥ 55 years with CKD stages 3 and 4, attending a secondary prevention program during 2012–2013.

**Design:**

The design of the study is a cross-sectional study of simple random sampling, and 308 patients were invited to participate.

**Setting:**

Patients were being treated in a CKD secondary prevention program in Bogotá, Colombia, during 2012–2013.

**Patients:**

Participants were over 54 years diagnosed with CKD in stages 3 to 4 according to the K/DOQI classification.

**Measurements:**

CI was assessed using NEUROPSI and modified Lawton Scale; depression was measured with Yesavage Geriatric Depression Scale and the MINI International Neuropsychiatric Interview.

**Methods:**

Through an interview with the subjects, information regarding age, occupation, civil status, educational level, and clinical baseline variables was collected. Clinical assessment with specific instruments was performed by a multidisciplinary team composed of nephrologists, a psychiatrist, a neurologist, and a neuropsychologist.

**Results:**

Two hundred and fifty-one patients agreed to participate. The average age was 76.3 (SD = 7.9) years, 67 % were males, and 86.5 % had CKD stage 3. Overall prevalence of CI was 51 % (95 % CI 44.7 to 57.2), and the prevalence of major depression reached 8 % (95 % CI 4.5 to 11.3); 4.8 % of the patients (*n* = 12) had both CI and depression.

**Limitations:**

A limitation of the study is its design, which does not allow establishing the direction of the association between predictors and outcomes. Suggested associations must be interpreted cautiously as they are generated as hypothesis, which should be investigated in properly designed trials.

**Conclusions:**

CI and depression are prevalent conditions among patients with CKD stages 3–4, with the greatest occurrence of CI, affecting half of the investigated Colombian patients with age ≥ 55 years.

## Abrégé

**Mise en contexte:**

On a constaté un lien entre la croissance de la population âgée et l’augmentation de pathologies comorbides telles que les maladies rénales chroniques et la dépression ou un trouble cognitif. Ce dernier peut varier de la déficience légère observée lors du vieillissement normal à un trouble cognitif léger, dont la prévalence s’étend de 1 à 29 % chez les gens âgés de plus de 65 ans, jusqu’à une déficience grave dont la prévalence se situe entre 6 et 42 %. Le trouble cognitif provoqué par la dépression a des répercussions sur les performances fonctionnelles des personnes âgées.

**Objectifs de l’étude:**

Cette étude avait pour but de qualifier la prévalence d’un trouble cognitif et de la dépression chez les patients âgés de plus de 55 ans atteints d’insuffisance rénale chronique (IRC) de stade 3 ou de stade 4, et engagés dans un programme de prévention secondaire en 2012 et 2013.

**Type d’étude:**

Il s’agit d’une étude transversale où on a utilisé un échantillonnage aléatoire simple et à laquelle 308 patients ont été invités à participer.

**Participants et cadre de l’étude:**

L’étude a porté sur des cas de patients provenant d’un programme de prévention secondaire à Bogota, en Colombie. Les participants étaient tous âgés de plus de 54 ans et avaient reçu un diagnostic d’IRC de stade 3 ou de stade 4 conformément au classement de la KDOQI (*Kidney Disease Outcome Quality Initiative*).

**Mesures:**

Le trouble cognitif a été mesuré à l’aide de l’évaluation neuropsychologique NEUROPSI et d’une échelle de Lawton modifiée. La dépression a été diagnostiquée en utilisant l’échelle de dépression gériatrique de Yesavage et le questionnaire MINI (*Mini International Neuropsychiatric Interview*).

**Méthodologie:**

Les renseignements personnels des participants tels que l’âge, la profession, l’état civil, le niveau de scolarité et les paramètres cliniques initiaux des participants ont été recueillis par le biais d’entrevues. L’évaluation clinique, effectuée à l’aide d’instruments spécifiques, est le fruit de la collaboration d’une équipe multidisciplinaire composée de néphrologues, d’un psychiatre, d’un neurologue et d’un neuropsychologue.

**Résultats:**

En tout, 251 patients ont accepté de participer à l’étude. Les hommes constituaient 67 % de la cohorte, l’âge médian se situait à 76,3 ans avec un écart-type de 7,9 ans et 85 % des participants souffraient d’IRC de stade 3. La prévalence globale d’un trouble cognitif était de 51 % (IC à 95 % entre 44,7 et 57,2) et la prévalence de dépression majeure se situait à 8 % (IC à 95 % entre 4,5 et 11,3). De plus, 4,8 % des participants (n = 12) présentaient à la fois un trouble cognitif et une forme de dépression.

**Limites de l’étude:**

La méthodologie de cette étude en limite la portée puisqu’elle ne permet pas d’établir la nature de l’association entre les indicateurs prévisionnels et les résultats. Ainsi, les liens potentiels devraient être interprétés avec prudence. Ils sont générés à titre d’hypothèses et devraient faire l’objet d’études ultérieures menées dans le cadre d’essais bien conçus.

**Conclusions:**

La dépression et les troubles cognitifs sont des affections prévalentes chez les patients souffrant d’IRC de stade 3 et de stade 4. Les troubles cognitifs se sont avérés très fréquents, affectant la moitié des patients Colombiens de plus de 55 ans ayant fait l’objet de cette étude.

## What was known before

There is scarcity of data of the prevalence of cognitive impairment and major depression in the population of patients with early stages of chronic kidney disease.

## What this adds

We found a high prevalence of cognitive impairment and 8 % of major depression in this population. These findings may be of prognostic significance and may have therapeutic implications.

## Background

Growth of the elderly population is associated with an increased rate of comorbid conditions such as chronic kidney disease (CKD) and cognitive impairment (CI), a precursor of dementia and, in turn, a key factor for the treatment of patients with CKD [1]. CKD and CI have common risk factors, which include advanced age, high blood pressure (HBP), diabetes mellitus (DM), and dyslipidemia, all of them clearly associated with vascular disease. CKD itself turns into a risk factor for the development of cerebrovascular disease, which is associated with the development of CI and depression, probably mediated by small vessel cerebrovascular disease [2–4].

An association between impairment of the glomerular filtration rate (GFR) and an increase in CI has been found [5–8]. CI is a clinical condition ranging from minimal cognitive deficit such as may be seen with normal aging to mild cognitive impairment (MCI) and overt dementia. The most frequent causes of CI are Alzheimer’s disease or cerebrovascular disease [9]. The prevalence of MCI ranges from 1 to 29 % in people over 65 years of age, and in 12 % of whom, there is a risk that the condition progresses into dementia within a year [10]. The rate of moderate to severe CI ranges between 6 and 42 %, depending on the type of instruments used to assess it, while the rate of dementia in people over 65 years of age reaches 10 % and increases with age [11]; the prevalence of Alzheimer’s disease combined with vascular dementia is 34 % [12–14].

According to Alexopoulos, cerebrovascular disease may predispose, trigger, and pertetuate a depressive syndrome in the elderly [15, 16]; besides it may produce CI which can affect the functional capacity and leading to the hypothesis of a common substrate for both [17].

CKD is an increasingly prevalent disease, particularly among people over 60 years, a population at high risk for CI [18]. The elderly CKD population represent 50 % of new cases of end-stage kidney disease (ESKD) in the USA [19, 20].

The purpose of this study is to describe the prevalence of CI and depression in a population with CKD stages 3–4 attending a secondary prevention program in Colombia.

## Methods

Following approval from the Fundación Universitaria de Ciencias de la Salud Medical School, the San Jose Hospital Ethics in Research Committee, and the RTS Ethics Committee, a descriptive, cross-sectional study was undertaken. Participants were patients over 54 years of age according with previous studies (6); attending a CKD secondary prevention program in Bogotá, Colombia, during 2012–2013; diagnosed with CKD in stages 3 to 4 according to the K/DOQI classification [21] (GFR 30–59.9 and 15–29.9 ml/min, respectively); with at least two nephrologist visits in the program; and with at least one estimation of the GFR. Patients who provided informed consent where included in the study. Patients with signs or symptoms of uremia and those with visual impairment impeding the application of the neuropsychological tests were excluded.

By interview, information regarding age, occupation, civil status, and educational level was collected. Clinical information concerning previous personal history (stroke, hypothyroidism, smoking, Diabetes Mellitus type 2 (DM2), anemia) in addition to data such as body mass index (BMI) using Quetelet’s formula (i.e., weigh/height^2^), cause of renal disease (DM2, HBP, glomerulopathy, obstructive nephropathy), alcohol consumption (over five drinks per week), and the last estimation of glomerular filtration rate (eGFR) were obtained from the clinical record.

Clinical assessment with specific instruments was performed by a multidisciplinary team composed of nephrologists, a psychiatrist, a neurologist, and a neuropsychologist.

### Instruments

Instruments chosen to be applied in the present study were selected due to simple and rapid application as well as appropriate sensitivity and specificity.

### Neuropsychological assessment

#### Short Neuropsychological Instrument in Spanish (NEUROPSI)

NEUROPSI is a short neuropsychological assessment specifically developed for Spanish-speaking population, which has been validated in Mexico and may be applied to people with a low educational level or illiterate subjects. The overall repeated test reliability is 0.87, and its sensitivity is 91.5 % [22]. NEUROPSI assesses specific cognitive domains including orientation, attention, concentration, language, memory, visual motor skills, executive functions, reading, writing, and computing. The maximal score is 130. Result interpretation is adjusted for the age and educational level of the subject and is reported as follows: normal, mild disturbance, moderate disturbance, and severe disturbance.

### Psychiatric assessment

#### Yesavage

The short version (15 questions) of the Geriatric Depression Scale by Yesavage was applied. The maximal score is 15, and the result may be reported as normal (0–5), moderate depression (6–9), or severe depression (10–15). Sensitivity and specificity of the Spanish validated version are 81.1 and 76.7 %, respectively, for a cutoff point ≥ 5 [23, 24].

#### International Neuropsychiatric Interview (MINI 5.0.0) by Sheehan and Lecrubier

MINI is a short structured diagnostic interview exploring the main psychiatric disturbances from axis I from the DSM-IV and the CIE-10 [25, 26]. Results from several studies have shown that MINI has high validity and reliability scores and it may be applied in a much shorter time (mean 18.7 ± 11.6 min, median 15 min) compared to other instruments (e.g., the SCDI-P mentioned in the DSM-III-R and the CIDI, a structured interview aimed at non-clinical interviewers, developed by the World Health Organization for the CIE-10) [26, 27]. In this study, MINI was applied by an experienced psychiatrist; as it is known, this improves the operating characteristics of the test (sensitivity 96 %, specificity 88 %, positive predictive value 87 %, negative predictive value 97 %) [25–27].

### Assessment of activities of daily living (ADL)

#### Modified Lawton score, validated in Spanish by Vergara et al. for being applied to older people

The Lawton ADL Scale is a test used to assess functional and physical impairment in patients with dementia and specifically evaluates factors from the onset of disease. The test was applied to patients whose cognitive condition allowed so; otherwise, it was responded with the help of the caregiver. Fourteen instrumental activities of daily living (IADL) in total were evaluated [28, 29]. A score > 7 at the time of the interview was considered as a significant functional impairment or a dementia syndrome. Other disease conditions may alter or reduce the specificity of the test [28, 29].

### Operative variables definitions

#### Cognitive impairment

As per Petersen’s criteria [30], CI was defined as follows:

A. Normal (N): normal result in NEUROPSI and no significant impairment in Lawton’s test (≤ 7).

B. Mild cognitive impairment (MCI): mild impairment as per NEUROPSI result and no significant impairment in Lawton’s test (> 7).

C. Moderate cognitive impairment: mild or moderate impairment as per NEUROPSI result and significant impairment in Lawton’s test (> 7).

D. Severe cognitive impairment: severe impairment as per NEUROPSI result and significant impairment in Lawton’s test (> 7).

#### Depression

Major depression: Patients with a score > 9 in the Yesavage test and a depression diagnosis as per MINI or patients with a score > 5 and a severe depression diagnosis as per MINI were defined as having major depression. Dysthymia was excluded.

### Sample

The sample size was calculated using the TAMAMU program® [31] Simple random sampling from the registry of patients with CKD stages 3–4 was performed. For estimating the prevalence of CI, an expected proportion of 30 % [32], a 5 % maximal difference, and a 5 % type I error were used. For estimating the prevalence of depression, the expected proportion was 23 % [33], type I error was 5 %, and maximal difference was 5 %. For estimating the prevalence of CI and depression, 257 and 224 patients, respectively, were required. On the largest sample size, a 20 % adjustment for non-response rate was performed. Consequently, a final sample size of 308 patients was obtained.

### Statistical analysis

Qualitative variables were summarized by descriptive statistics with absolute and relative frequencies; quantitative variables were reported using central tendency and dispersion measures. The prevalence of CI and depression is reported as a proportion and 95 % confidence intervals (95 % CI).

Two logistic regression models were undertaken on an exploratory basis; the first one for assessing factors associated with CI and the second for assessing factors associated with depression. All the demographic and clinical variables were examined using backward stepwise logistic regression analysis, and values of *p* < 0.05 were considered to be statistically significant. All statistical tests of hypothesis are two-sided. Statistical analysis was made with Stata 13.0 ®.

## Results

Among the 308 patients invited to participate, 251 accepted. Mean age was 76.3 (SD = 7.9) years, 67 % of the patients were males, the median of schooling years was 6 (IRQ = 7), 58 % of the interviewed patients were married, and 53 % had retired. All socio-demographic characteristics of the subjects are shown in Table 1.

**Table 1:** Demographic characteristics of the population (*n* = 251)

	CI no (*n* = 123)		CI yes (*n* = 128)		Total (*n* = 251)		*p* value
	*n*	%	*n*	%	*n*	%	
Age, years, mean (SD)	75.3	(7.2)	77.4	(8.6)	76.4	(7.9)	0.023
Males, *n* (%)	86	(69.9)	82	(64.1)	168	(66.9)	0.324
Females, *n* (%)	37	(30.1)	46	(35.9)	83	(33.1)	
Years of school attendance							
None	4	(3.3)	3	(2.3)	7	(2.8)	0.000
1–4 years	30	(24.4)	28	(21.9)	58	(23.1)	
5–9 years	32	(26.0)	71	(55.5)	103	(41.0)	
≥ 10 years	57	(46.3)	26	(20.3)	83	(33.1)	
Occupation, *n* (%)							
Current worker	16	(13.0)	14	(10.9)	30	(12.0)	0.037
Retired	74	(60.2)	59	(46.1)	133	(53.0)	
Home	32	(26.0)	54	(42.2)	86	(34.3)	
Unemployed	1	(0.8)	1	(0.8)	2	(0.8)	
Civil status, *n* (%)							
Single	20	(16.3)	9	(7.0)	29	(11.6)	0.011
Living with a partner	5	(4.1)	6	(4.7)	11	(4.4)	
Married	72	(58.5)	63	(49.2)	135	(53.8)	
Widowed	23	(18.7)	46	(35.9)	69	(27.5)	
Divorced	3	(2.4)	4	(3.1)	7	(2.8)	

Regarding medical history, 83 % of the patients had HBP, 50 % were either overweight or obese, 30 % had hypothyroidism, and 24 % DM; 27 % of the patients were smokers and 9 % had suffered a cerebrovascular event; and 86.5 % had CKD stage 3 with an estimated mean GFR of 42 ml/min (interquartile range, IQR = 14). The most frequent cause of CKD was HBP (68 %). (See Table 2.)

**Table 2:** Clinical characteristics of patients with CKD 3 and 4

Clinical characteristics	Cognitive impairment no		Cognitive impairment yes		Total		*p* value
	(*n* = 123)		(*n* = 128)		(*n* = 251)		
Previous medical history, *n* (%)							
High blood pressure (HBP)							
Slight HBP	81	(65.9)	75	(58.6)	156	(62.2)	0.606
Moderate HBP	20	(16.3)	23	(18.0)	43	(17.1)	
Severe HBP	3	(2.4)	5	(3.9)	8	(3.2)	
Without HBP	19	(15.4)	25	(19.5)	44	(17.5)	
Diabetes mellitus, *n* (%)	29	(23.6)	31	(24.2)	60	(23.9)	0.905
Hypothyroidism, *n* (%)	34	(27.6)	41	(32.0)	75	(29.9)	0.377
Stroke, *n* (%)	10	(8.1)	12	(9.4)	22	(8.8)	0.742
Smoking, *n* (%)	33	(26.8)	35	(27.3)	68	(27.1)	0.927
Anemia, *n* (%)	–	–	4	(3.1)	4	(1.6)	–
Sedentary lifestyle, *n* (%)	64	(52.0)	72	(56.3)	136	(54.2)	0.417
Alcohol consumption^b^, *n* (%)	8	(6.5)	6	(4.7)	14	(5.6)	0.541
Glomerular filtration rate, ml/min, median (IQR)	44	(37–49)	39.5	(31–48)	42	(34–48)	0.028
Stage 3 chronic kidney disease, *n* (%)	110	(89.4)	107	(83.6)	217	(86.5)	0.177
Stage 4 chronic kidney disease, *n* (%)	13	(10.6)	21	(16.4)	34	(13.5)	
Cause of kidney disease, *n* (%)							
Diabetes	21	(17.1)	27	(21.1)	48	(19.1)	0.750
HBP	89	(72.4)	81	(63.3)	170	(67.7)	
Glomerulonephritis	1	(0.8)	4	(3.1)	5	(2.0)	
Obstructive condition	6	(4.9)	10	(7.8)	16	(6.4)	
Unknown	6	(4.9)	6	(4.7)	12	(4.8)	
Body mass index, kg/mt^2^, median (IQR)^a^	24	(23–27)	25	(22–27)	25	(23–27)	0.908
Normal	58	(47.5)	60	(49.2)	118	(48.4)	0.726
Overweight	53	(43.4)	48	(39.3)	101	(41.4)	
Obese	11	(9.0)	14	(11.5)	25	(10.2)	

*IQR* interquartile range

^a^Data were available for 244 patients

^b^World Health Organization criteria

Overall prevalence of CI was 51 % (95 % CI, 44.7–57.2); 36 % of the patients had MCI and 15 % had moderate or severe CI. Among CKD stage 3 patients, prevalence of MCI was 36 % and that of moderate and severe CI was 13.4 %. Among CKD stage 4 patients, prevalence of MCI was 35.3 % and that of moderate and severe CI was 26.5 %. Multivariate analysis showed that preservation of glomerular filtration reduces the rate of CI (OR, 0.97; 95 % CI, 0.95–0.99; *p* = 0.008). Age was related with higher frequency of CI (OR, 1.01; 95 % CI, 1.004–1.027; *p* = 0.007; see Table 4).

Prevalence of major depression was 8 % (95 % CI, 4.5–11.3). In CKD stage 3 patients, depression prevalence was 8.3 % while it was 5.9 % in those in CKD stage 4; the detailed results of diagnostic test used for CI and depression are showed in Table 3. A multivariate analysis showed that living with a partner was the most significant protective factor for depression (see Table 4). Twelve patients (4.8 %) had both CI and major depression.

**Table 3:** Cognitive impairment and depression by diagnostic test used

Classification	(*n* = 251)		(*n* = 251)	
Cognitive impairment, *n* (%)	NEUROPSI test		Diagnosis^a^	
Normal	123	(49.0)	123	(49.0)
Mild	51	(20.3)	90	(35.8)
Moderate	51	(20.3)	24	(9.6)
Severe	26	(10.4)	14	(5.6)
Depression, *n* (%)	Yesavage test		Diagnosis^b^	
Normal (≤ 9)	237	(94.4)	231	(92.0)
Major depression (> 9)	14	(5.6)	20	(8.0)

^a^Diagnosis in CI: NEUROPSI plus Lawton plus evaluation by neurologist

^b^Diagnosis in depression: Yesavage plus MINI

**Table 4:** Multivariate analysis for risk of cognitive impairment and depression

	OR	(95 % CI)	*p* value
Cognitive impairment^a^			
Glomerular filtration rate	0.97	(0.95–0.99)	0.008
Age	1.01	(1.004–1.027)	0.007
Major depression^b^			
Living with a partner	0.34	(0.13–0.90)	0.029
Age	0.97	(0.96–0.98)	0.000

^a^Adjusted by glomerular filtration rate, age, sex, diabetes, high blood pressure, years of school attendance, and body mass index (kg/mt^2^)

^b^Adjusted by glomerular filtration rate, age, sex, living with a partner, diabetes, hypothyroidism, and years of school attendance

## Discussion

In the present study of patients with stages 3–4 CKD, we found a prevalence of CI with 35.9 % of them with MCI. These results are lower than those presented by Tangri, in which a prevalence rate of 77 % of MCI was reported for patients with < 30 ml/min of eGFR [34]. Our lower prevalence could be partly explained because our population was primarily composed of stage 3 patients (86.5 %) compared to stage 4 patients [34]. The high prevalence of CI is important as it impacts patient self-care capacity, quality of life, and treatment adherence, all aspects that are difficult to discern on routine evaluation.

The 51 % prevalence of CI in stages 3 and 4 is similar to patients ≥ 70 years of age (which is similar to mean age in the present study) participating in the 1999–2004 National Health and Nutrition Examination Survey (NHANES). In NHANES, the prevalence of CI ranged between 46.3 and 46.8 % depending on the method used for estimating GFR: the MDRD study equation or CKD EPI equation, respectively [1]. In the present study, the mean patient age was similar to NHANES and the GFR was estimated by MDRD 4 equation. The marginal difference may have resulted from the difference in study populations as our study focused on patients in a renal clinic whereas NHANES was a general population cohort.

The only meta-analysis evaluating the impact of CKD on CI included 54,779 subjects and examined both cross-sectional and prospective studies [35]. They reported a clear association between CI in patients with CKD vs. patients without CKD (OR 1.65, 95 % CI 1.32–2.05; *p* < 0.001 and OR 1.39, 95 % CI 1.15–1.68; *p* < 0.001, respectively).

The present study is comparable to previous cross-sectional studies in design; however, it is the first in a South American population [35, 36].

Few trials to date (3) have used complex test batteries consisting of ≥ 6 tests that explore multiple cognitive domains. NEUROPSI, which was the test applied in our patients, explores eight domains and is reliable in the detection of CI. Furthermore, NEUROPSI was originally designed in Spanish which improves applicability compared to other neuropsychological tests like MOCA or MMSE. The high prevalence of CI in CKD suggests CKD is a significant, independent factor in CI and has not traditionally been used in the development of CI screening tools [37].

We used a unique combination of the NEUROPSI and Lawton tests as well as a neurologist evaluation to classify mild CI. This approach give us a more specific qualification of CI and allowed us to differentiate mild from more advanced CI based on the compromise of living activities. To assess depression, we used the Yesavage and the MINI to increase the specificity of diagnosis and exclude other types of depressive pictures like dysthymia or depressive reactions.

As compared to previous studies, the prevalence of major depression was low in our study. Some studies such as the one by Newman [38] were undertaken in patients with dementia and report a 3.2 % major depression prevalence in those with Alzheimer’s disease and 21.2 % in those with vascular dementia. Other studies, such as the one by Agganis, included patients undergoing hemodialysis and report significant depression symptoms in 23.7 % of the patients according to CES-D [33]. Lopes defines depression as the presence of depressive symptoms during the previous year as per the clinical record and self-reported by the patient within the last 4 weeks (20 %) [39]. Cukor found a 20 % rate of chronic depression and anxiety as per SCID-I and BDI [40]. Among the depressed patients, in 17, it was a de novo condition, while in 3, it was recurrent. Furthermore, and like in the Cukor study, most of our patients corresponded to late depression which is usually associated with cerebrovascular disease.

The strengths of this study include being the first one exploring the presence of CI and depression in CKD patients not undergoing dialysis in Latin America. Despite a 21 % non-response ratio, the sample size for estimating the prevalence of such conditions in the studied population was attained. Patients were directly assessed by specialists.

A limitation of the study is its design, which does not allow establishing the direction of the association between predictors and outcomes. Suggested associations must be interpreted cautiously as they are generated as hypothesis, which should be investigated in properly designed trials.

Given the high prevalence of CI, carrying out a cognitive assessment of patients entering CKD prevention programs is recommended and would allow earlier detection and interventions for preventing progression.

## Conclusions

Cognitive impairment and depression are prevailing conditions in CKD patients, cognitive impairment being the most frequent, and affecting about half of the patients.
